# Ultraviolet disinfection robots to improve hospital cleaning: Real promise or just a gimmick?

**DOI:** 10.1186/s13756-020-00878-4

**Published:** 2021-02-12

**Authors:** Magda Diab-El Schahawi, Walter Zingg, Margreet Vos, Hilary Humphreys, Lorena Lopez-Cerero, Astrid Fueszl, Jean Ralph Zahar, Elisabeth Presterl, for the ESCMID Study Group on Nosocomial Infections “The decontamination research working group”

**Affiliations:** 1 Department of Infection Control and Hospital Epidemiology, Medical University of Vienna, Waehringer Guertel 18-20, 1090 Vienna, Austria; 2 Infection Control Programme, University Hospital of Zurich, Zurich, Switzerland; 3 Department of Medical Microbiology and Infectious Diseases, Erasmus MC University Medical Center Rotterdam, Rotterdam, The Netherlands; 4 Department of Clinical Microbiology, Royal College of Surgeons in Ireland, Dublin, Ireland; 5 Department of Microbiology, Beaumont Hospital, Dublin, Ireland; 6 Microbiology Unit, Hospital Virgen Macarena, Sevilla, Spain; 7 Unité de contrôle et de prévention du risque infectieux, service de microbiologie, groupe hospitalier universitaire, Hôpital Avicenne, Paris Seine Saint-Denis, France; 8 UFR-SMBH, Université Paris XIII, Paris Sorbonne, France

**Keywords:** UV-disinfection, Infection control, Disinfection robots

## Abstract

The global COVID-19 pandemic due to the novel coronavirus SARS-CoV-2 has challenged the availability of traditional surface disinfectants. It has also stimulated the production of ultraviolet-disinfection robots by companies and institutions. These robots are increasingly advocated as a simple solution for the immediate disinfection of rooms and spaces of all surfaces in one process and as such they seem attractive to hospital management, also because of automation and apparent cost savings by reducing cleaning staff. Yet, there true potential in the hospital setting needs to be carefully evaluated. Presently, disinfection robots do not replace routine (manual) cleaning but may complement it. Further design adjustments of hospitals and devices are needed to overcome the issue of shadowing and free the movement of robots in the hospital environment. They might in the future provide validated, reproducible and documented disinfection processes. Further technical developments and clinical trials in a variety of hospitals are warranted to overcome the current limitations and to find ways to integrate this novel technology in to the hospitals of to-day and the future.

## Background

Bacteria and viruses survive on inanimate surfaces in hospitals for up to several days and longer [1, 2]. The global COVID-19 pandemic due to the novel coronavirus SARS-CoV-2 has challenged the availability of disinfectants [3]. Shortages in surface disinfectants, although worrying, is not the only aspect that matters in providing clean environments in healthcare. Dancer and colleagues showed that only 50% of surfaces in hospital rooms are sufficiently cleaned between patients stays [4]. Thus, the hospital environment is a possible source for the transmission of pathogens in the healthcare environment [5].

In China the COVID-19 pandemic has stimulated the production of ultraviolet (UV)-disinfection robots by companies and institutions. Yet, there is little information about their operational details [6]. “Disinfection robots” such as the UVD robotic device manufactured by UVD Robots ApS, Tru-D SmartUVC manufactured by Lumalier or cleaning robots manufactured by the Shanghai-based RMiRob [7, 8] are increasingly advocated as a simple solution for the immediate disinfection of rooms and spaces of all surfaces in one process. Disinfection robots seem attractive to hospital management, mainly because of automation and apparent cost savings by reducing cleaning staff.

The idea of a self-contained device or system for cleaning and disinfection in hospitals may be gaining momentum. However, even if only applicable for disinfection, such devices prompt discussion on their added benefit.

## Description of the technology and the devices

Robots may be defined as machines programmed by humans to perform tasks and navigate themselves through space and time on their own. The most widely applied technology focusses on surface disinfection by applying ultraviolet (UV)-C radiation. All types of UV-disinfection robots offer a non-touch technology, delivering disinfection by irradiation of effective intensity to kill microorganisms, but with no mechanical removal of dirt or biological material, which contain bacteria and viruses.

Ultra violet light at a wavelength of 254 nm (UV-C) is bactericidal, sporicidal, fungicidal and virucidal [9]. Shadowing with UV-C light, where some surfaces are not exposed due to obstruction or inaccessibility, is a known limitation of this type of technology [9]. Shadowing and distance significantly reduces UV-C intensity and thus limit an efficient disinfection process. The current literature indicates that UV-C disinfection systems can efficiently reduce microbial contamination in in vitro settings [7, 9]. In practice, this has also been shown for ambulances [10], for patient rooms [5, 11] and for bacterial contamination in operating rooms [12]. Efficacy is a function of the initial inoculum, soiling, applied energy and time of exposure [13]. These vary depending upon the microorganism and in case of bacteria, whether it is in a vegetative state or spore [9]. Most importantly, UV disinfection systems must be validated for each room or setting before use, and be supervised after initial deployment. Defining the exact UV-C device positions for clinical settings is critical to ensure the proper functioning of a UV-C device to achieve the anticipated disinfection efficacy.

## Advantages of UVC disinfection robots

As its antimicrobial activity is well described UV-C can represent a valuable alternative to solution-based products in times of limited supply of traditional surface disinfectants [9]. Manual cleaning and disinfection is variable because efficacy hugely depends on individuals and their motivation, and assessing this requires direct on-site observation. Despite best practice recommendations, manual cleaning in each hospital is based on local protocols, training, understanding, renewal and staff turnover of cleaning staff, as well as the control and the inspection of their performance. Evidence further suggests that manual cleaning and disinfection are often inadequate and result in residual contamination [4]. Besides killing microorganisms on surfaces, disinfection robots offer reproducibility by recording automatically the operation parameters of the disinfection process and by this, can provide quality assurance. Therefore, automated disinfection could allow the validation of the disinfection process with reproducible and documented disinfection results.

Advantages of UV-C robots are: (1) Robotic disinfection will work in an unmanned and standardized fashion, without the need for ongoing human presence at the disinfection site. Therefore, exposure of health care workers to harmful UV radiation can be avoided during the process [7]. (2) Applying UV-C as a final disinfection step after manual cleaning and manual disinfection provides an additional hygiene benefit to reducing cross-transmission and healthcare associated infections [8]. (3) UV light does not leave any residues, making this an environmental friendly disinfection method.

## Limitations

Turnaround times for single rooms in hospitals need to be short, given high bed occupancy levels in many countries. UV-C robots will need additional time that interferes with daily hospital routines. Thus their use must be integrated in to the workflow of hospitals. This new technology is best used to supplement current hospital cleaning and disinfecting practices. Dirt and organic soiling are the biggest challenges to the effective use of these robots because UV-C does not deliver sufficient energy to inactivate bacterial and viral pathogens embedded in such material. Thus, manual cleaning is a prerequisite for the use of UVC disinfection, which needs staff and additional time. Moreover, disinfection robots need an expert supervisor for setting and overseeing the programme, and to reset after encountering unforeseen obstacles. Using a disinfection robot like a vacuum cleaner, in addition to routine measures adds work instead of exploiting its full potential [10].

Today’s hospital designs and inventory are not built to allow disinfection robots to meet their potential. Ideally, disinfection robots would communicate with the environment for automated operability, being capable of entering patient rooms independently through electronic doors, detect if a patient room is still occupied, and turn on and off as a function of their position towards surfaces to be disinfected, and distance to an individual. In addition, unplanned cluttering of patient rooms and wards creates shadowing and limits robots navigating in space and reaching surfaces to be disinfected. The design and inventory of future hospitals must take into account electronic cross-talk with various systems of workflow and patient care of which cleaning and disinfection robots will be part. Planners and future architects should integrate robotic disinfection in their structural design.

## Conclusions

The current COVID-19 pandemic boosts innovation on many public, societal and medical levels and disinfection practices are not an exception. Disinfection robots are a promising tool for surface decontamination in the hospital already today, but with even greater potential tomorrow. Further design adjustments of hospitals and devices are needed to overcome the issue of shadowing and free the movement of robots in the hospital environment. One-size does not fit all, and apart from communication between robot and the environment, more work must also be invested in defining efficient wavelength and exposure time to allow sufficient energy to be applied on each surface, as a function of the intended pathogen to be inactivated. Finally, a fit-for-purpose hospital environment would allow disinfection robots to function independently.

Presently, disinfection robots do not replace routine (manual) cleaning but may complement it. They might in the future provide validated, reproducible and documented disinfection processes. Further technical developments and clinical trials in a variety of hospitals are warranted to overcome the current limitations and to find ways to integrate this novel technology in to the hospitals of to-day and the future.
